# Rapid Quantitation of Anatoxins in Benthic Cyanobacterial Mats Using Direct Analysis in Real-Time–High-Resolution Tandem Mass Spectrometry

**DOI:** 10.1021/acs.est.2c05426

**Published:** 2022-09-20

**Authors:** Daniel G. Beach, Meghann Bruce, Janice Lawrence, Pearse McCarron

**Affiliations:** † Biotoxin Metrology, National Research Council Canada, 1411 Oxford Street, Halifax, Nova Scotia B3H 3Z1, Canada; ‡ Canadian Rivers Institute, University of New Brunswick, P.O. Box 4400, Fredericton, New Brunswick E3B 5A3, Canada; § Department of Biology, University of New Brunswick, 10 Bailey Drive, Fredericton, New Brunswick E3B 5A3, Canada

**Keywords:** ambient ionization, DART−HRMS, cyanotoxins, high-throughput screening, Microcoleus, Phormidium

## Abstract

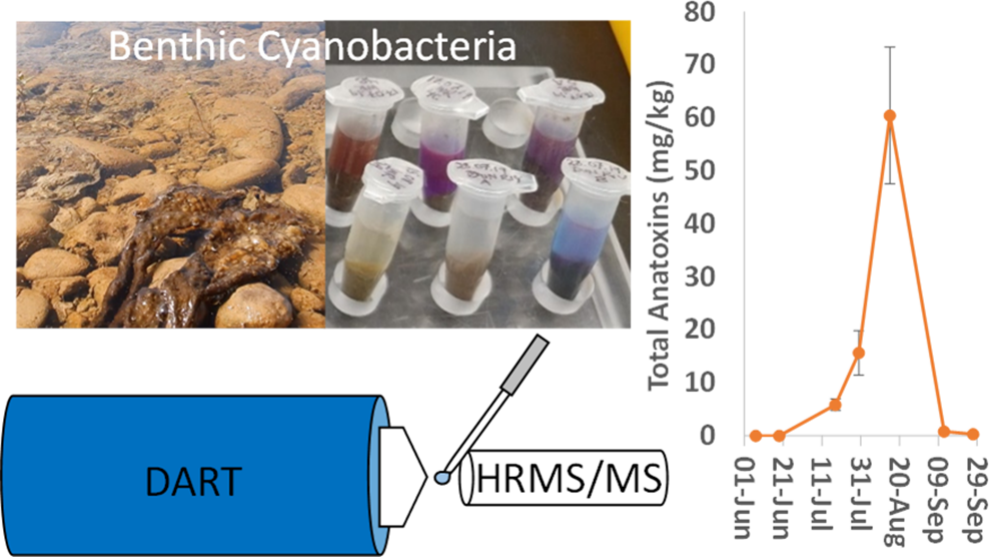

Toxic benthic cyanobacterial mats are increasingly reported worldwide as being responsible for animal mortalities due to their production of the potent neurotoxin anatoxin-a (ATX) and its analogues. Improved analytical methods for anatoxins are needed to address public health and watershed management challenges arising from extremely high spatial and temporal variability within impacted systems. We present the development, validation, and application of a direct analysis in real-time–high-resolution tandem mass spectrometry (DART–HRMS/MS) method for analysis of anatoxins in cyanobacterial field samples, including a simplified sample preparation approach. The method showed excellent sensitivity and selectivity for ATX, homoanatoxin-a, and dihydroanatoxin-a. Isotopically labeled ATX was used as an internal standard for all three analogues and successfully corrected for the matrix effects observed (86 ± 16% suppression). The limit of detection and recovery for ATX was estimated as 5 ng/g and 88%, respectively, using spiked samples. The total analysis time was ∼2 min, and excellent agreement was observed with results from a liquid chromatography–HRMS reference method. Finally, the DART–HRMS/MS method was applied to a set of 45 *Microcoleus*-dominated benthic cyanobacterial mat samples from the Wolastoq near Fredericton, Canada, demonstrating its power and applicability in enabling broad-scale field studies of ATX distribution.

## Abstract 2

A rapid DART−HRMS/MS method was developed, validated, and applied to study the highly variable levels of anatoxins in *Microcoleus*-dominated benthic cyanobacterial mats from the Wolastoq near Fredericton, Canada.

## 1.0 Introduction

Cyanobacteria are globally ubiquitous but can cause environmental and human health problems when they multiply to high cell concentrations in the aquatic environment. This can include either dense surface blooms or the proliferation of benthic cyanobacterial mats, which grow on rocks, sediment, or macrophytes on the bottoms of lakes or rivers. There are increasing reports of toxic benthic cyanobacterial proliferations worldwide, which are often associated with animal deaths caused by the potent neurotoxin anatoxin-a (ATX).^1−5^ In many cases, toxicity can be attributed to cyanobacteria of the genera *Microcoleus*, *Phormidium,* or *Oscillatoria*, which are morphologically similar and poorly resolved by genetics and taxonomy.^1,6^

There are several challenges associated with studying, monitoring, and assessing risks associated with benthic ATX-producing cyanobacteria. These include analytical challenges such as the low molecular weight, high polarity, and poor stability of ATX, the complex assemblages of cyanobacteria and other microorganisms found in benthic microbial mats, as well as the overall high spatial and temporal variability in mat occurrence and ATX concentration.^1,7−9^

**Figure 1:**
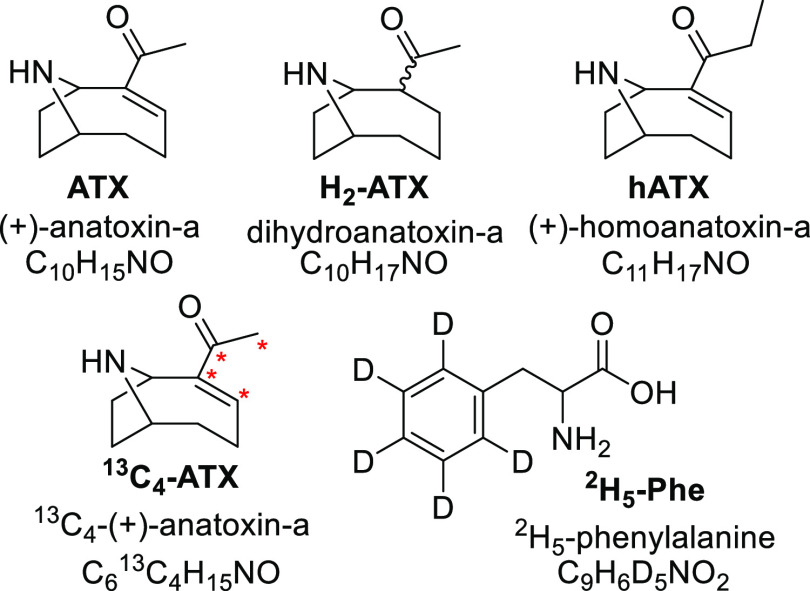
Structures and formulae of anatoxins and internal standards analyzed in this study. Asterisks indicate sites of ^13^C labeling on ^13^C_4_-ATX.

ATX typically exists in the environment as a mixture with other structural analogues, usually homoanatoxin-a (hATX) and dihydroanatoxin-a (H_2_-ATX), collectively referred to as anatoxins (ATXs, Figure 1). ATXs can be analyzed using a variety of chemical and biochemical techniques, with liquid chromatography–mass spectrometry (LC–MS)^10−16^ being the most common for quantitative analysis. Immunochemical and receptor binding assays are also commercially available.^17,18^ High-resolution mass spectrometry (HRMS) is particularly effective at resolving ATX from its common isobaric interference phenylalanine, which can cause interference and ionization suppression in some MS-based methods.^16,19−21^ The improved selectivity of HRMS over low-resolution MS instruments has also enabled the use of direct ionization techniques for ATX analysis including direct analysis in real time (DART),^22^ matrix-assisted laser desorption ionization,^20^ and laser-induced thermal desorption ionization.^21,23^ These techniques offer the potential for higher throughput than LC–MS because of their lack of chromatographic separation and reduced requirement for sample preparation but also pose challenges in selectivity and matrix effects. Recently, excellent selectivity and sensitivity were demonstrated for analysis of ATX and hATX by full-scan DART–HRMS in cultured cyanobacteria with a limit of detection (LOD) of 1 ng/mL and excellent quantitative agreement with LC–HRMS.^22^ However, the significant impact of matrix effects on accuracy (50% suppression) and high sample-to-sample variability (30% RSD) of the method, which used single-point matrix-matched calibration, were highlighted as limitations in need of further study.

In the summers of 2018 and 2019, dogs died of acute neurological symptoms after spending time on the shore of the Wolastoq (Saint John River) near Fredericton, New Brunswick, Canada.^24^ Despite the fact that this area had not previously been associated with significant cyanobacterial blooms, ATXs were identified in samples associated with the poisonings, and a widespread proliferation of benthic cyanobacterial mats was discovered in the area. This has led to increased study of the drivers of mat occurrence and toxicity, as well as highlighting the challenges of monitoring and managing this emerging problem in the region, all of which have emphasized the need for rapid and reliable analytical methods for ATXs.

Here, we describe the development and validation of a DART–HRMS/MS method for the analysis of ATXs in complex cyanobacterial mat field samples. This includes refinement of our earlier full-scan method^22^ to gain the selectivity needed for analysis in a complex cyanobacterial mat field sample matrix using HRMS/MS, the quantitation of another common ATX analogue, H_2_-ATX, and mitigation of matrix effects using an isotopically labeled ATX standard. We also present the development of a simple sample preparation method for cyanobacteria that is amenable to higher throughput analysis. Throughout method development and validation, we relied on a highly sensitive LC–HRMS reference method for ATXs to gain confidence in the performance of the DART–HRMS/MS method. Finally, we applied the developed method to a set of cyanobacterial mat samples from the Wolastoq in order to investigate spatial and temporal trends and intermat variability of ATXs in a real system.

## 2.0 Experimental Section

### 2.1 Chemicals and Reagents

A certified reference material calibration solution for ATX (NRC CRM-ATX-a) and an in-house calibration solution for hATX^25^ were obtained from the National Research Council Canada (Halifax, Nova Scotia). Standards of ^13^C_4_-(+)-anatoxin-a (^13^C_4_-ATX) and H_2_-ATX were purchased from Eurofins Abraxis (Warminster, PA, USA).

Methanol and acetonitrile (Optima LC–MS grade) were from Fisher (Ottawa, ON, Canada). Formic acid (LC–MS grade) and L-phenyl(^2^H_5_)alanine were from Sigma-Aldrich (Oakville, ON, Canada). Deionized water was produced by passing distilled water through a Milli Q Reference A+ System (Millipore, Bedford, MA, USA).

### 2.2 Cyanobacterial Mat Sample Collection and Preparation

Initial method development was carried out on a set of 10 cyanobacterial samples that included benthic mats collected in Nova Scotia in 2019^26^ and samples of *Microcoleus-*dominated mats collected in the summers of 2019 and 2020 from the Wolastoq (Saint John River) near Fredericton, New Brunswick (coordinates in Table S1). For validation using LC–HRMS, the sample set was expanded with 35 additional samples of the cyanobacterial mat and their associated sediment collected along the Wolastoq during the summers of 2018, 2019, and 2020.

Samples were collected in 15 mL sterile centrifuge tubes, maintained at ambient temperatures, and homogenized within 24 h of collection using 15 mL disposable tissue grinders (VWR International, Radnor, PA, USA). Once ground, homogenized samples were stored at −20 °C prior to further processing.

Prior to analysis, subsamples of thawed homogenate (∼1 mL) were centrifuged at 21 000 *g* at 4 °C for 20 min. A subsample of the supernatant (100 μL) was then transferred to a 0.22 μm Innosep PVDF spin filter (Canadian Life Sciences, Peterborough, ON, Canada), immediately mixed with 100 μL of 120 ng/mL ^13^C_4_-ATX in methanol and centrifuged at 6720 *g* for 5 min at room temperature, and analyzed by DART–HRMS/MS and LC–HRMS. This filtration step was only required in cases where samples were also to be run by LC–HRMS as DART–HRMS/MS did not require samples to be filtered.

### 2.3 DART–HRMS

All experiments were carried out on a Q Exactive HF Orbitrap mass spectrometer (Thermo, Waltham, MA, USA). A DART-SVP source was coupled to the mass spectrometer using a Vapur interface (Ionsense, Saugus, MA). Optimized DART–HRMS parameters included the use of He as the DART gas, a DART temperature of 350 °C, a capillary temperature of 200 °C, a max IT fill time of 300 msec, and an S-Lens RF level of 50 (arbitrary units). MS data were collected simultaneously in full-scan (FS) mode with a *m*/*z* 150 to 250 mass range, as well as tandem mass spectrometry (MS/MS) for precursor ions of *m*/*z* 166.1 (ATX), 168.1 (H_2_-ATX), 180.1 (hATX), and 170.1 (^13^C_4_-ATX), using the parallel reaction monitoring scan mode with a collision energy (CE) of 15 V, a 0.4 *m*/*z* isolation window, and the 30 000 resolution setting. Product ions used for quantitation were the [M + H − NH_3_]^+^ ions for ATX, hATX, and ^13^C_4_-ATX at *m*/*z* 149.0961, 163.1117, and 153.1095, respectively, as well as the [M + H – C_2_H_3_N]^+^ ion at *m*/*z* 125.0961 for H_2_-ATX.

Samples were introduced by pipetting 5 μL of the extract onto the tip of a Dip-it sampling rod (Ionsense) held manually, approximately midway between the DART source and the ceramic interface tube for 10 s. All samples were analyzed in triplicate and a mixed standard (43 ng/mL ATX, 46 ng/mL hATX, 37 ng/mL H_2_-ATX, and 60 ng/mL ^13^C_4_-ATX) was run approximately every 10 samples.

Peak areas for quantitation were integrated manually in Xcalibur 4.0 software from chronograms of product ion *m*/*z* extracted with ± 5 ppm mass windows. Calibration of ATX was carried out by single-point double-isotope dilution using the ratio of ^13^C_4_-ATX:ATX in the spiked sample and standard, as follows
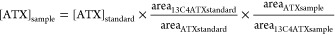
1where [ATX]_sample_ is the concentration of ATX in the field sample and [ATX]_standard_ is the ATX concentration in the neat ATX standard.

The concentrations of hATX and H_2_-ATX were determined by single-point external calibration corrected with the suppression factor for ^13^C_4_-ATX in each sample as in the following example for hATX
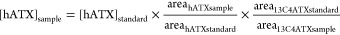
2where  is the suppression factor for a particular field sample.

### 2.4 LC–HRMS Analysis

LC–HRMS analyses were performed using an Agilent 1200 LC system (Agilent, Santa Clara, CA, USA) coupled to the mass spectrometer described above with a HESI-II heated ESI interface (ThermoFisher Scientific, Waltham, MA, USA). All LC separations were performed with an injection volume of 1 μL using an HSS T3 1.8 μm C_18_ column (100 × 2.1 mm; Waters, Milford, MA, USA) held at 40 °C with mobile phases A and B of H_2_O and acetonitrile, respectively, both containing 0.1% v/v formic acid. The elution gradient (0.2 mL/min) included a linear increase from 2 to 11% B over 25 min and then to 95% B over 0.1 min, followed by a 4.9 min hold at 95% B, with re-equilibration at 2% B for 5 min. HRMS data was acquired in positive ion mode using a combined FS and targeted MS/MS method. FS data were collected from *m*/*z* 100 to 500 using the 30 000 resolution setting, an AGC target of 1 × 10^6^, and a max IT of 100 ms. MS/MS spectra were acquired as for DART–HRMS/MS described above.

Calibration for ATX in LC–HRMS analysis was carried out by single-point double-isotope dilution, as described above for DART–HRMS. Homoanatoxin-a and H_2_-ATX were measured by external calibration. When signal intensity allowed, a 100-fold dilution with 1:1 MeOH/H_2_O was carried out to minimize matrix effects in LC–HRMS. Two H_2_-ATX diastereomer peaks at different retention times with MS/MS spectra matching that of H_2_-ATX were observed in LC–HRMS, as reported previously,^15,27^ and were summed in both standards and samples.

## 3.0 Results and Discussion

### 3.1 DART–HRMS/MS Method Development

The original goal of this work was to apply a recently reported DART–HRMS method, which was developed for screening ATXs in laboratory cultures of cyanobacteria,^22^ to benthic cyanobacterial mat samples from the Wolastoq in New Brunswick, Canada. However, benthic cyanobacterial mats are highly complex, both in terms of the biological community and the chemical matrix present in sample extracts, which has previously led to problems in analytical selectivity in some methods.^5^ Throughout the study, results from DART analysis of field samples were compared to those obtained using an LC*–*HRMS reference method in order to assess selectivity and accuracy. The LC*–*HRMS method has a LOD of 0.1 ng/mL in culture extracts and excellent resolution of the ATXs from each other (Figure S1) and potential matrix interferences such as phenylalanine (Phe).^22^ In order to evaluate the suitability of the FS DART method for analysis of ATXs in a more complex matrix, a preliminary set of 10 cyanobacterial field samples was analyzed by both DART*–*HRMS and LC–HRMS. This analysis showed that in several cases, high concentrations of ATXs were determined by DART*–*HRMS in the absence of detection of ATXs by LC*–*HRMS (Figure S2). This was particularly true for hATX but also for ATX and H_2_-ATX in some samples, even when using the maximum FS resolution setting of 240 000. The differences observed were attributed to poor selectivity, making the previous DART*–*HRMS method unsuitable for the analysis of ATXs in field samples.

The additional selectivity of MS/MS was therefore investigated as a way of improving method performance. Product ions and CE used for quantitation were chosen experimentally based on selectivity and sensitivity. While low-mass product ions (e.g., C_3_H_6_N^+^ ions at *m*/*z* 56.0495 for ATX and H_2_-ATX) showed the highest overall signal intensity at moderate CE (Figure S3), these showed relatively poor selectivity compared with higher mass product ions at lower CE. A CE of 15 V was therefore chosen for all further experiments, with product ion spectra for ATX, hATX, H_2_-ATX, and ^13^C_4_-ATX in standards and samples shown in Figure S4. No significant advantage in selectivity was observed by increasing the resolution setting in MS/MS beyond the minimum setting of 15 000 in test samples, but since a setting of 30 000 did not show any disadvantage in MS cycle time, it was used going forward. Excellent selectivity between ATX and its common interference Phe was also observed in HRMS/MS (Figure S5), where no significant signal was observed from Phe for the *m*/*z* 149.0959 product ion used for quantitative analysis of ATX by DART*–*HRMS/MS. Using this DART*–*HRMS/MS method, excellent agreement was observed with LC*–*HRMS in all cases for the preliminary sample set (Figure S2).

As found previously for culture extracts,^22^ sample introduction by pipetting 5 μL of liquid sample onto a Dip-it sampling rod and manually introducing it into the space between the DART source and the MS inlet for 10 s was found to be simple and effective. No impact on the DART peak area was observed with small deviations of positioning between the DART source and the MS inlet, although holding the sampling rod steady was important for achieving a good DART peak shape. While potentially improving method automation, spotting of samples and standards onto steel mesh sampling cards (Open-Spot) consistently showed reduced ATX sensitivity when a new card was used compared to cards that had been used previously, even though sample carryover was ruled out as a possible cause.

In order to facilitate method throughput, a simple extraction procedure was developed that required only sample homogenization, cell lysis, and centrifugation. This was possible due to the high moisture content in the majority of mat samples. After sample homogenization, cell lysis by freeze/thaw and centrifugation, the resulting supernatant, consisting primarily of interstitial liquid from the mats and cell contents, could be analyzed directly by DART without filtration. No significant difference in sensitivity or precision was observed between aqueous cyanobacterial mat extracts and those containing 50 or 75% methanol; however, samples that contained methanol evaporated slightly more rapidly, resulting in sharper DART peaks. Samples of the supernatant were therefore diluted 1:1 with MeOH prior to analysis.

The use of an isotopically labeled internal standard was investigated in order to improve method precision and accuracy. Deuterated phenylalanine (^2^H_5_-Phe), an internal standard previously reported for the analysis of ATX using laser diode thermal desorption,^21^ was first investigated for use with DART. However, when spiked into a series of field sample extracts at 100 ng/mL, the ^2^H_5_-Phe signal was almost completely suppressed, and therefore, it was not useful as an internal standard with DART (Figure S6).

An isotopically labeled ATX standard (^13^C_4_-ATX) was therefore used to develop a double-isotope dilution calibration approach for quantitation of ATX in both LC–HRMS and DART–HRMS/MS (eq 1). Unlike in LC–HRMS, where ATXs are separated chromatographically and detected at different retention times with different mobile-phase compositions and coeluting matrix compounds, both of which affect ionization efficiency, all analytes in DART–HRMS are ionized simultaneously. This meant that ^13^C_4_-ATX could also confidently be used to effectively correct for the suppression observed for hATX and H_2_-ATX by applying a suppression factor determined from the ratio of ATX response between standard and each sample to the results of external calibration (eq 2). For quantitative screening of field samples, a spike concentration of 60 ng/mL was chosen to match WHO guidelines for short-term exposure to ATX in recreational waters.^28^ To further simplify the sample preparation method, sample spiking was done by making a 120 ng/mL stock solution of ^13^C_4_-ATX in MeOH, which was used to carry out the 1:1 dilution of mat lysate samples.

Using this approach, results were obtained in units of μg/mL of mat lysate, but for many applications, it may be more desirable to obtain a mass fraction value for ATXs in benthic cyanobacterial mats. Given their high moisture content, it was reasoned that μg/mL concentrations in mat lysates were similar to mg/kg wet weight mass fractions in the mats themselves. In order to establish the equivalence of these units, 15 mat samples were homogenized and split into two subsamples, with one-half being weighed and extracted with 50% MeOH and the other half being centrifuged and analyzed directly after spiking, as described above. An excellent correlation with a slope of 1.0 was observed between the two sample preparation approaches (Figure S7), with a 16 ± 12% absolute difference in ATX measured in ng/mL and ng/g (average ± standard deviation, *N* = 15). This was considered acceptable for the intended application of rapid quantitative screening, and concentrations are therefore reported as mg/kg wet weight going forward based on the described lysate procedure.

**Figure 2:**
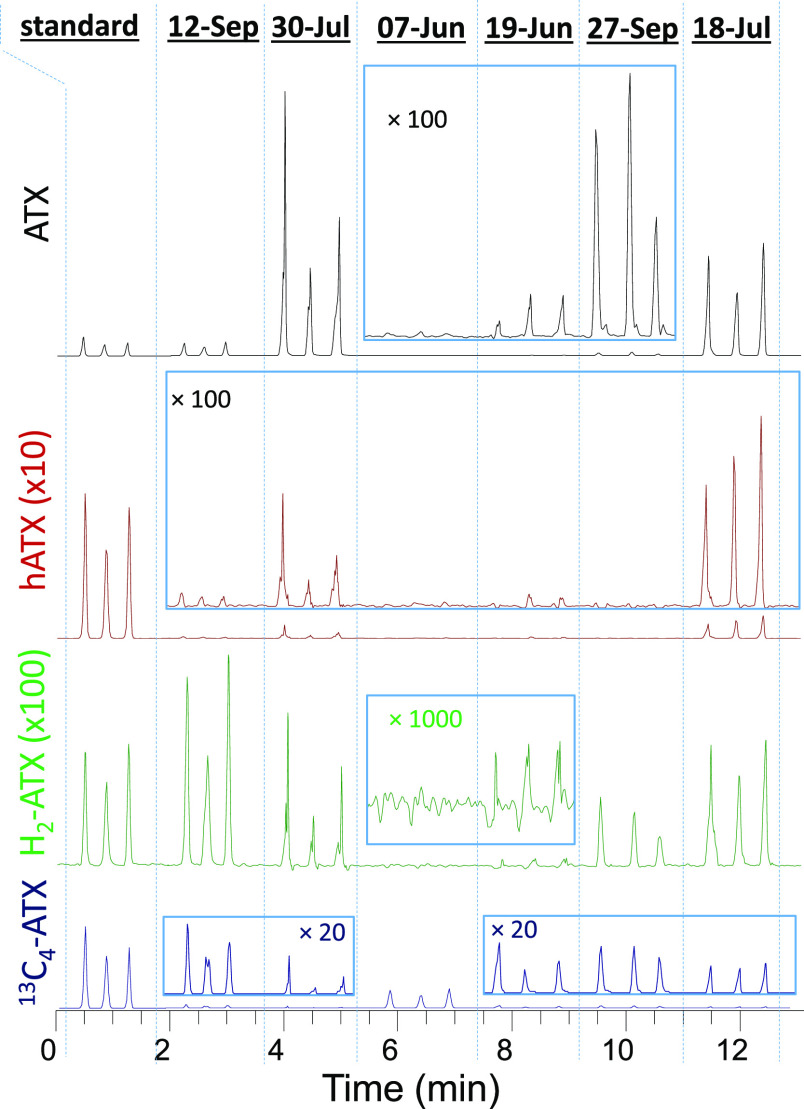
Extracted ion chronograms from DART–HRMS/MS showing triplicate analysis of a mixed anatoxin standard and six benthic cyanobacterial mat samples collected from site 3 on the Wolastoq in 2019 spiked with 60 ng/mL ^13^C_4_-ATX. Traces show extracted product ion *m*/*z* of 149.0961, 163.1117, 125.0961, and 153.1095 ± 5 ppm for ATX, hATX, H_2_-ATX, and ^13^C_4_-ATX, respectively.

Chronograms showing triplicate analysis of a mixed ATX standard and a selection of ^13^C_4_-ATX spiked mat samples from site 3 on the Wolastoq (Table S1) are shown in Figure 2. The variability in the sensitivity of ^13^C_4_-ATX between samples, which was spiked at 60 ng/mL in all samples and standards, demonstrated the wide range in signal suppression observed between samples in DART–HRMS, which was however effectively corrected using the internal standard.

### 3.2 Quantitative Capabilities of DART–HRMS/MS

The primary benefit of DART is the rapid analysis time, but it was also evident that the DART–HRMS/MS method also possesses quantitative capabilities beyond those of a qualitative screening method. The typical quantitative figures of merit were thus investigated and are compared to those of the LC–HRMS reference method (Table 1). Matrix-matched and neat ATX, hATX, and H_2_-ATX calibration curves were analyzed by both methods. Estimated LODs for LC–HRMS were based on the signal-to-noise ratio (S/N) of low-level standards spiked into a pooled sample of cyanobacterial mat sample extracts that were negative for ATXs by LC–HRMS, with the LOD, defined as S/N = 3. These same samples were also analyzed by DART–HRMS/MS, but the LOD was defined as the concentration at which a signal of 3× the background detected in the pooled control extract would be measured. This approach was chosen because of the variable signal background in DART–HRMS, hindering reliable calculations of S/N values. The LOD reported for H_2_-ATX (24 μg/kg) was significantly higher than that for ATX (4.8 μg/kg) and hATX (2.4 μg/kg), which can be attributed to lower sensitivity and higher chemical background observed in DART for the precursor–product ion combination used in H_2_-ATX quantitation. The LODs estimated from the spiked pooled control sample are directly related to the level of ionization suppression in that particular sample, which was relatively high at 96 ± 1% suppression (SD, *N* = 18). Because ^13^C_4_-ATX was spiked at 60 ng/mL into all of the real samples and standards in the study, it was also possible to assess the suppression observed in each individual sample, which was usually somewhat lower than the pooled control sample at 86 ± 16% RSD (*N* = 34) but significantly higher than in LC–HRMS at 19 ± 24% RSD (*N* = 33). The sample-specific suppression factors could further be used to estimate individual LODs for each sample, which varied significantly between 0.3 and 23 μg/kg with an average of 4.7 μg/kg for ATX. This approach could be useful in the future in establishing expected levels of detection in negative samples.

**Table 1:** Figures of Merit of the DART–HRMS/MS Method Compared to Those of the LC–HRMS Reference Method

figure of merit	toxin	LC–HRMS	DART–HRMS/MS
estimated LOD (μg/kg)	ATX	0.13	4.8
	hATX	0.11	2.4
	H_2_-ATX	0.15	24
precision (% relative standard deviation, *N* = 3)	ATX	4^a^	23 ± 11^b^
% suppression (*N* = 34)	ATX	19 ± 24	86 ± 16
linearity (*R*^2^)	ATX (0.14–86 ng/mL)	>0.9999	0.9999
	hATX (0.15–91 ng/mL)	>0.9999	0.9994
	H_2_-ATX (0.12–74 ng/mL)	>0.9999	0.9998
15 μg/kg spike recovery (*N* = 5)	ATX	83 ± 5	82 ± 11
	hATX	66 ± 4	102 ± 19
	H_2_-ATX	58 ± 1	83 ± 14
150 μg/kg spike recovery (*N* = 5)	ATX	83 ± 3	84 ± 15
	hATX	82 ± 6	101 ± 12
	H_2_-ATX	75 ± 3	76 ± 13
run time (min)	30 (single injection)	2 (triplicate analysis)	

a Percent relative standard deviation of triplicate analysis of ATX in a cyanobacterial reference material.^25^

b Average % relative standard deviation of triplicate analysis of ATX in 23 field samples.

**Figure 3:**
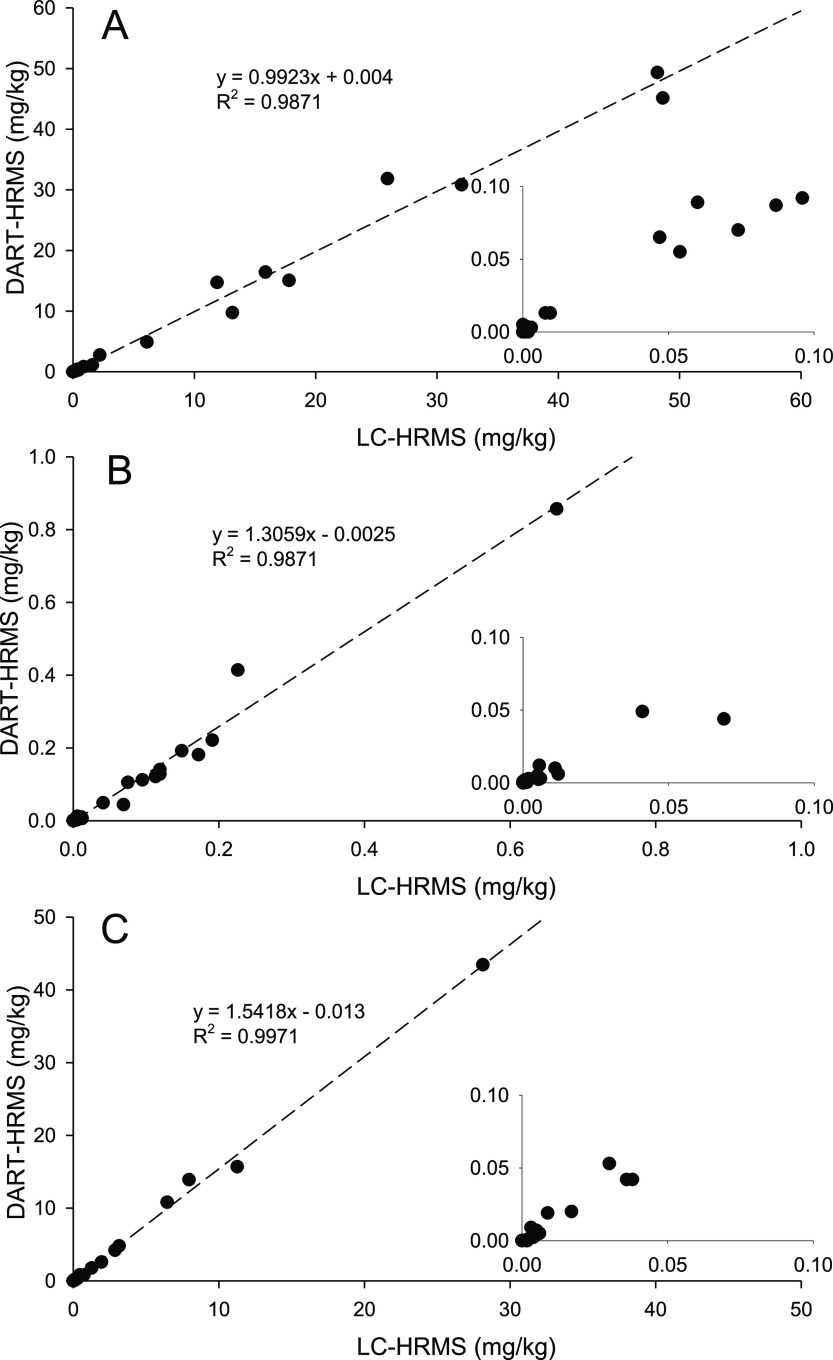
Quantitative analysis of anatoxin-a(A), homoanatoxin-a (B), and dihydroanatoxin-a (C) in 45 cyanobacterial mat samples by DART–HRMS/MS compared to those from LC–HRMS. Dashed lines show linear regression of each plot with the equations and coefficient of determination (*R*^2^) given. Low-level results (<0.1 mg/kg) are shown in the insets.

Method accuracy was assessed by spike recovery experiments (Table 1) and by comparing the results of real sample analysis by DART–HRMS/MS to those from LC–HRMS (Figure 3). Recovery was determined by spiking 0.4 g subsamples of five different negative field samples with 25 μL of a mixed standard of ATX, hATX, and H_2_-ATX to give concentrations of either 15 ng/g or 150 ng/g each and then extracting following the developed procedure. Overall method performance was assessed by comparing DART–HRMS/MS results from all 45 benthic cyanobacterial mat samples to those from LC–HRMS. For ATX, where double-isotope dilution was used for both methods, excellent recoveries of 82–84% were observed along with an excellent correlation between the two methods (slope = 0.99, *R*^2^ = 0.98). Good general agreement was also observed for hATX and H_2_-ATX, where a combination of external calibration and sample dilution was used in LC–HRMS calibration, but results from DART–HRMS/MS were consistently slightly higher than those from LC–HRMS for these analogues.

Only one false positive was observed by DART–HRMS, with 0.005 mg/kg ATX being detected in a sample showing no ATXs by LC–HRMS. In all cases, samples showing ATXs by LC–HRMS above the estimated DART LODs were found to contain ATXs by DART–HRMS, although some differences in detection were observed for individual analogues in very low-level samples. In the future, appropriate thresholds in precursor and product ion mass accuracy and product ion ratio could be assigned to ensure that results are appropriate for the quantitative requirements of a given study.

### 3.3 Applications of DART–HRMS/MS to Benthic Cyanobacterial Mat Field Samples

**Figure 4:**
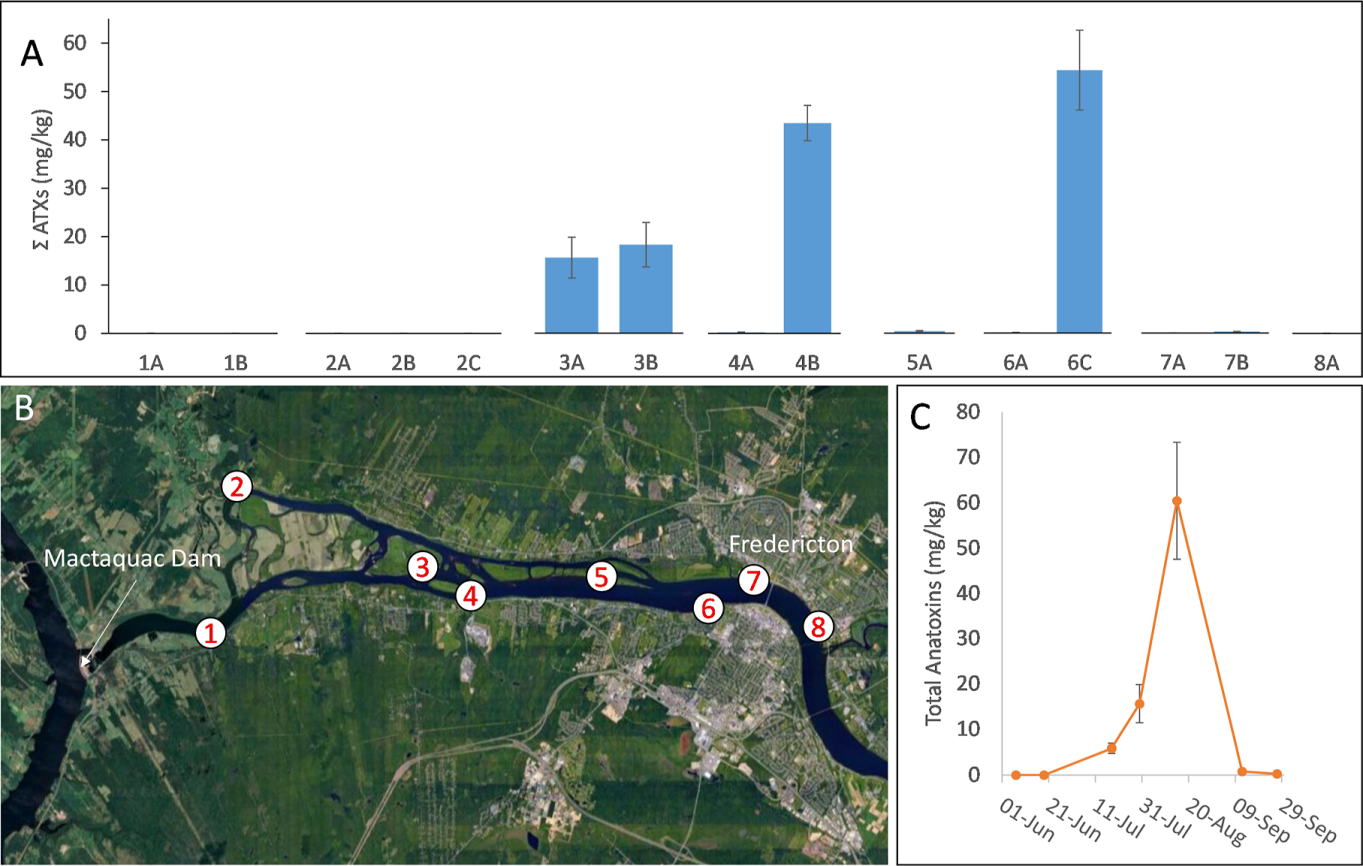
Spatial and temporal variability of total anatoxins in benthic cyanobacterial mat samples from the Wolastoq as measured by DART–HRMS/MS. Spatial variability (A) in samples collected on July 30th and 31st, 2019, where separate bars indicate individual samples taken from each site (B) and temporal variability (C) in samples collected from site 3 throughout the summer of 2019. Error bars show the standard deviation of triplicate analysis of a single sample.

The main motivation for this work was to develop an analytical method to enable a large-scale study of toxin distribution in benthic cyanobacterial mats. To demonstrate this application, DART–HRMS/MS results from 21 benthic cyanobacterial mats from the Wolastoq collected during a survey in the summer of 2019 were analyzed to study temporal and spatial trends as well as intermat variability (Figure 4). This included samples collected over a 2 day period in July 2019 (Figure 4A) at eight sites along the impacted section of the river between the Mactaquac Dam and the city of Fredericton, NB, Canada (Figure 4B), as well as samples collected from a single site over the course of the summer (Figure 4C). These results show high environmental variability, even between mat samples taken from the same site at the same time. This is consistent with observations from similarly impacted sites elsewhere^8^ and has been attributed to variability in the relative abundance of toxic genotypes.^29^ Despite this high within-site variability, a clear trend of increasing ATX concentrations peaking in mid-August followed by a drop to trace levels in September was observed at sample site 3 over the course of the summer. The results from DART–HRMS/MS and LC–HRMS analysis of the samples from Figure 4 are provided in Table S1 and show excellent agreement, further highlighting the suitability of DART for this type of environmental analysis.

The main advantages of DART–HRMS/MS were the rapid analysis (2 min/triplicate analysis) and simplified sample preparation. Limitations of the DART–HRMS/MS method included higher RSDs (on the order of 30% between replicate analyses) as well as higher and variable LOD when compared with LC–HRMS. However, considering the very high environmental variability and often high concentrations of ATXs observed in samples collected from the Wolastoq, these limitations are not viewed as significant for the current application.

The 2019 samples from the Wolastoq analyzed in this study were collected as part of an ongoing survey of benthic cyanobacterial mat distribution and population genetics. Since genetic analysis already requires cell lysis and sample homogenization, the addition of DART–HRMS/MS analysis was procedurally extremely simple, requiring only centrifugation and dilution of a subsample of supernatant with an internal standard. Future work will include the application of the DART–HRMS/MS method developed here to a broader study of the cyanobacterial mat and ATX occurrence in the Wolastoq. In the future, automation of sample introduction to DART would offer even greater improvements in sample throughput, further enabling large-scale studies of ATXs in large proliferations of benthic cyanobacterial mats.

## Supporting Information Available

Anatoxin concentrations, sampling dates, and sampling sites; extracted ion chromatograms from LC–HRMS; comparison between the LC–HRMS, DART–HRMS, and DART–HRMS/MS; energy resolved collision-induced dissociation of anatoxins; DART–HRMS/MS spectra of anatoxins; LC–HRMS/MS analysis of ATX and phenylalanine; DART–HRMS analysis of ^2^H_5_-Phe-spiked cyanobacterial samples; and equivalence of ng/mL and ng/g measurement of anatoxins (PDF)
