# Supplementary material for: Rapid Quantitation of Anatoxins in Benthic Cyanobacterial Mats Using Direct Analysis in Real-Time–High-Resolution Tandem Mass Spectrometry
Source: Environ Sci Technol. 2022 Sep 20;56(19):13837–44. doi: 10.1021/acs.est.2c05426 (PMC9535865; doi:10.1021/acs.est.2c05426)
Supplement: Supplementary file 1 — es2c05426_si_001.pdf [file es2c05426_si_001.pdf]

Supporting Information

Rapid Quantitation of Anatoxins in Benthic Cyanobacterial Mats Using Direct Analysis in Real Time–High Resolution Tandem Mass Spectrometry

Daniel G. Beach [a]*, Meghann Bruce[b], Janice Lawrence[c], Pearse McCarron[a]

[a] *Biotoxin Metrology, National Research Council Canada, 1411 Oxford Street, Halifax, Nova Scotia, B3H 3Z1, Canada*

[b] *Canadian Rivers Institute, University of New Brunswick, P.O. Box 4400, Fredericton, New Brunswick, E3B 5A3, Canada*

[c] *Department of Biology, University of New Brunswick, 10 Bailey Drive, Fredericton, New Brunswick, E3B 5A3, Canada*

*Corresponding author:
Dr. Daniel Beach
Tel.: +1 (902) 426-8274
E-mail address: daniel.beach@nrc-cnrc.gc.ca

Table of Contents **page #**

Table S1: Anatoxin concentrations, sampling dates and sampling sites S2
Figure S1: Extracted ion chromatograms from LC–HRMS S3
Figure S2: Comparison between the LC-HRMS, DART-HRMS and DART-HRMS/MS S4
Figure S3: Energy resolved collision induced dissociation of anatoxins S5
Figure S4: DART-HRMS/MS spectra of anatoxins S6
Figure S5: LC-HRMS/MS analysis of anatoxin-a and phenylalanine S7
Figure S6: DART-HRMS analysis of $^{2}H_{5}$-Phe spiked cyanobacterial samples S8
Figure S7: Equivalence of ng/mL and ng/g measurement of anatoxins S9

Table S1: Total anatoxin concentrations from cyanobacterial mat samples, sampling dates and coordinates of sampling sites on the Wolastoq.

Site Name (coordinates)	Date (2019)	Total ATXs[a] by DART-HRMS/MS (mg/kg ± SD, N = 3)	Total ATXs[a] by LC-HRMS (mg/kg)
NB-1 (45.958750, -66.827658)	Jul 31	0.016 ± 0.002	0.014
		<LOD	0.0003
NB-2 (45.992082, -66.819793)	Jul 30	0.0030 ± 0.0003	0.0049
		0.003 ± 0.001	0.0038
		<LOD	<LOD
NB-3 (45.974617, -66.759316)	Jun 7	<LOD	<LOD
	Jun 19	<LOD	0.0037
	Jul 18	6 ± 1	6.9
	Jul 30	16 ± 4	12
		18 ± 5	17
	Aug 15	60 ± 13	N/A[b]
	Sep 12	0.78 ± 0.07	0.76
	Sep 27	0.03 ± 0.05	0.032
NB-4 (45.967481, -66.745481)	July31	0.21 ± 0.08	0.18
		43 ± 4	33
NB-5 (45.973053, -66.698757)	Jul 30	0.4 ± 0.1	0.42
NB-6 (45.964856, -66.662421)	Jul 31	0.11 ± 0.03	0.078
		54 ± 8	52
NB-7 (45.972083, -66.647623)	Jul 31	0.013 ± 0.002	0.0092
		0.30 ± 0.03	0.26
NB-8 (45.961651, -66.627210)	Jul 31	0.023 ± 0.003	0.010

[a] Total ATXs is defined as the sum of anatoxin-a, homoanatoxin-a and dihydroanatoxin-a
[b] Sample not analyzed by LC-HRMS

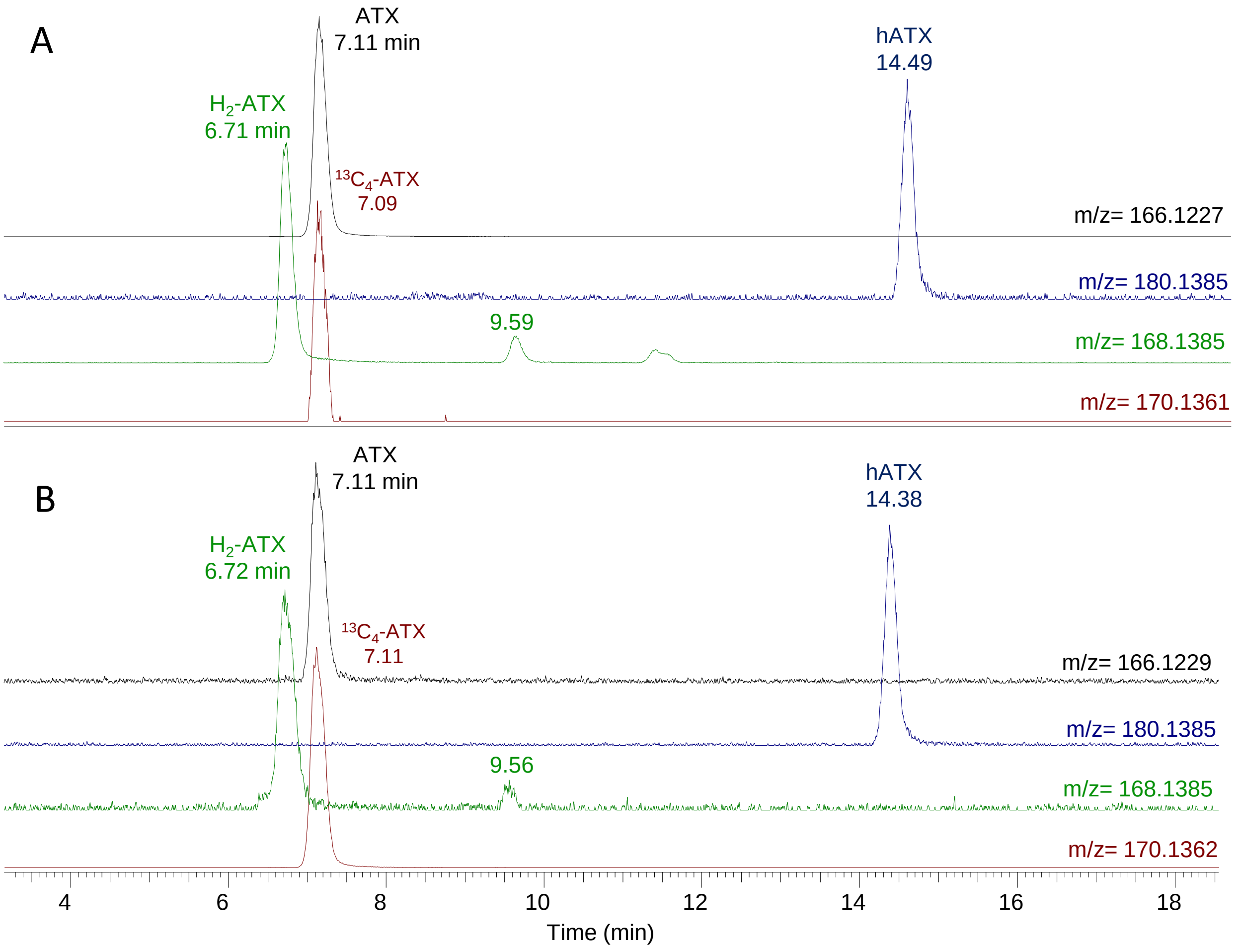

Figure S1: Extracted ion chromatograms from LC–HRMS analysis of a 100-fold dilution of an extract from a benthic cyanobacterial mat sample collected from site 6 on the Wolastoq on July 31st 2019 spiked with 60 ng/g $^{13}C_4$-anatoxin-a (A) and a mixed standard containing 7.0 ng/mL anatoxin, 7.0 ng/mL homoanatoxin, 7.2 dihydroanatoxin and 60 ng/mL $^{13}C_4$-anatoxin-a (B).

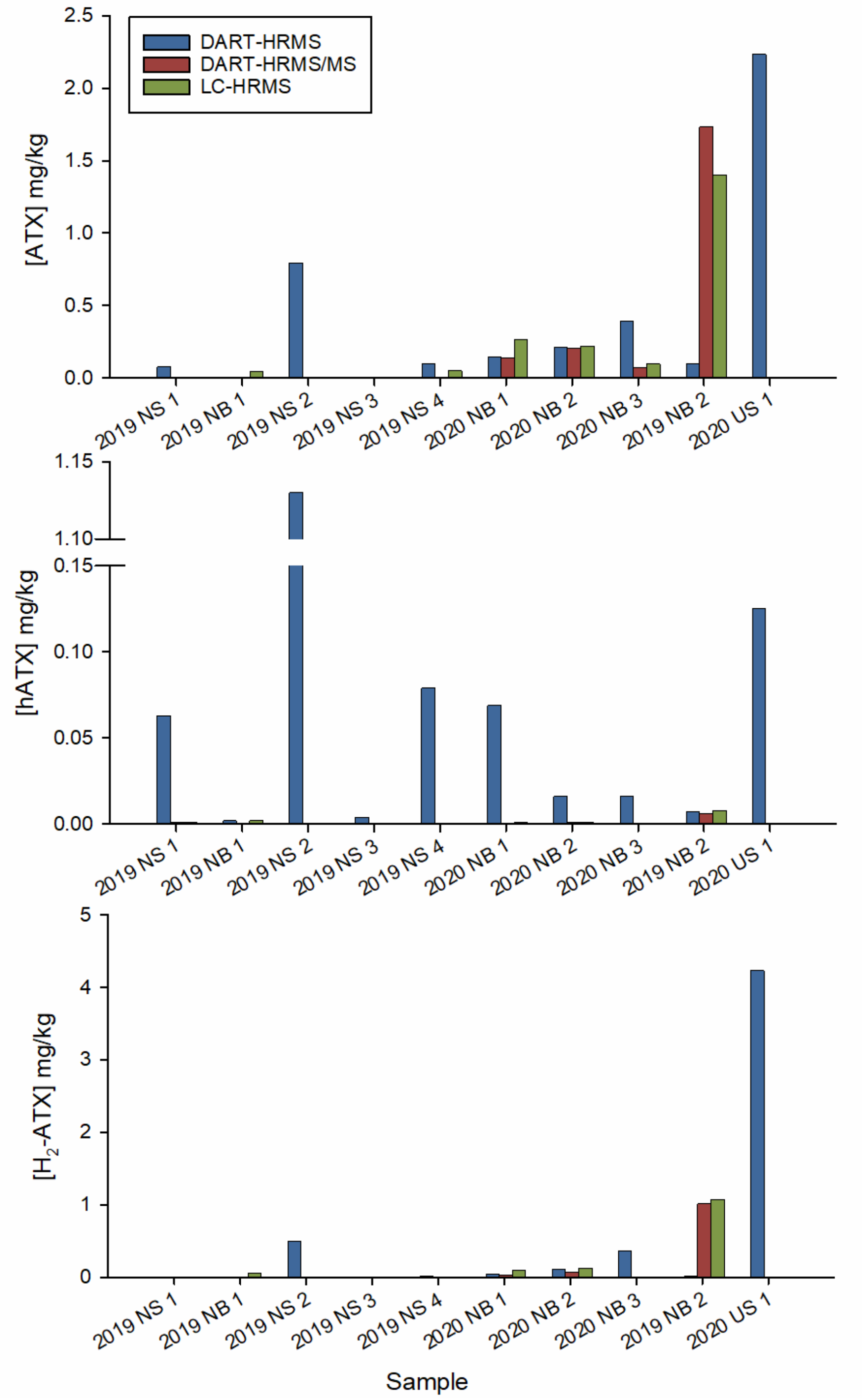

Figure S2: Selectivity comparison between the LC-HRMS reference method, DART-HRMS at the 240k resolution setting and DART-HRMS/MS for anatoxin-a (A), homoanatoxin-a (B) and dihydroanatoxin-a (C) in a preliminary set of cyanobacterial field samples.

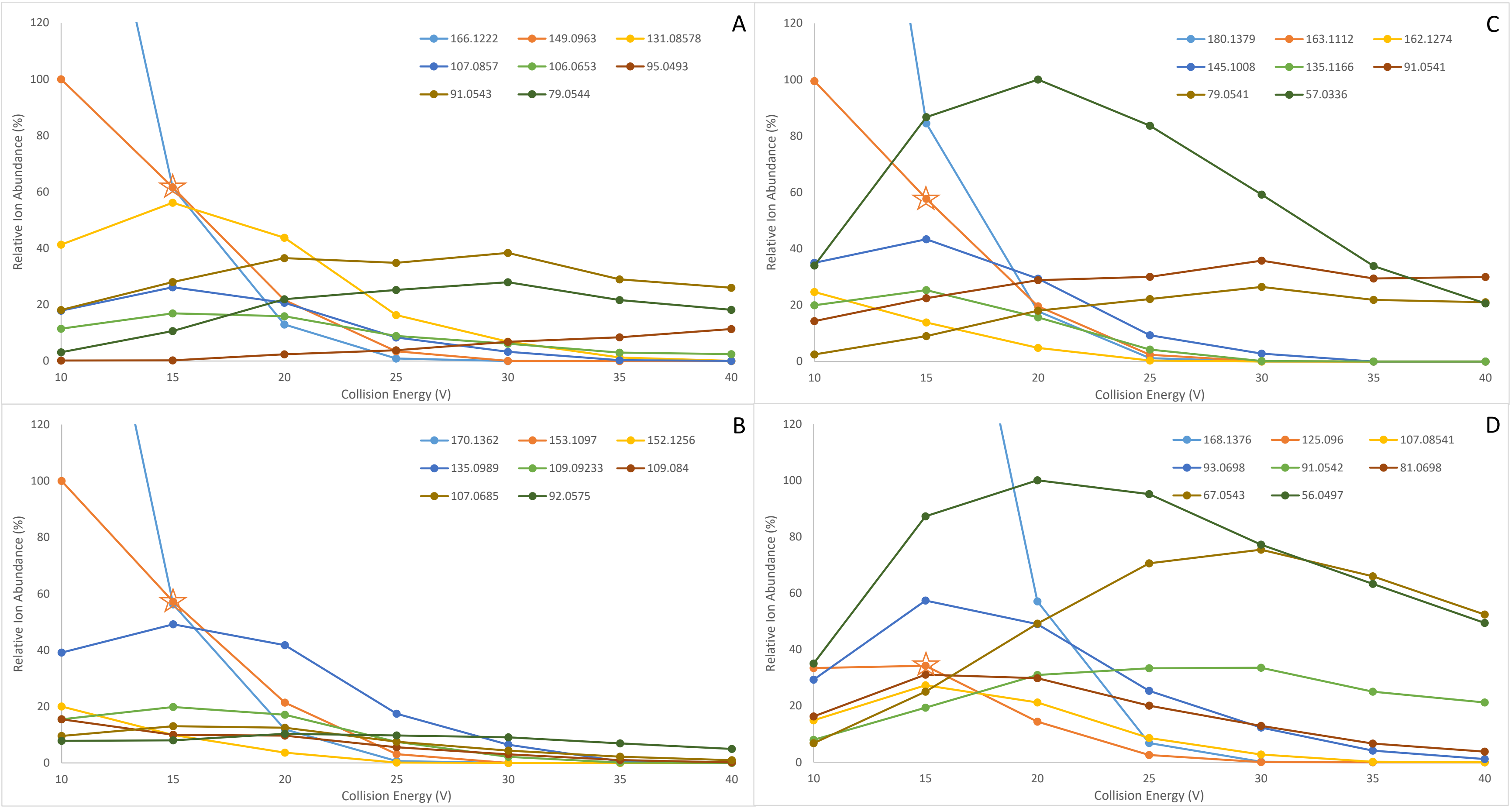

Figure S3: Energy resolved collision induced dissociation of anatoxin-a (A), $^{13}C_4$-anatoxin-a (B), homoanatoxin-a (C), dihydroanatoxin-a (D) showing how the relative abundance of the precursor (light blue) and major product ions, with the *m*/*z* indicated in the legend, vary with absolute collision energy in LC-HRMS/MS. The orange start on each plot indicates the product ion and collision energy chosen for quantitation of each compound.

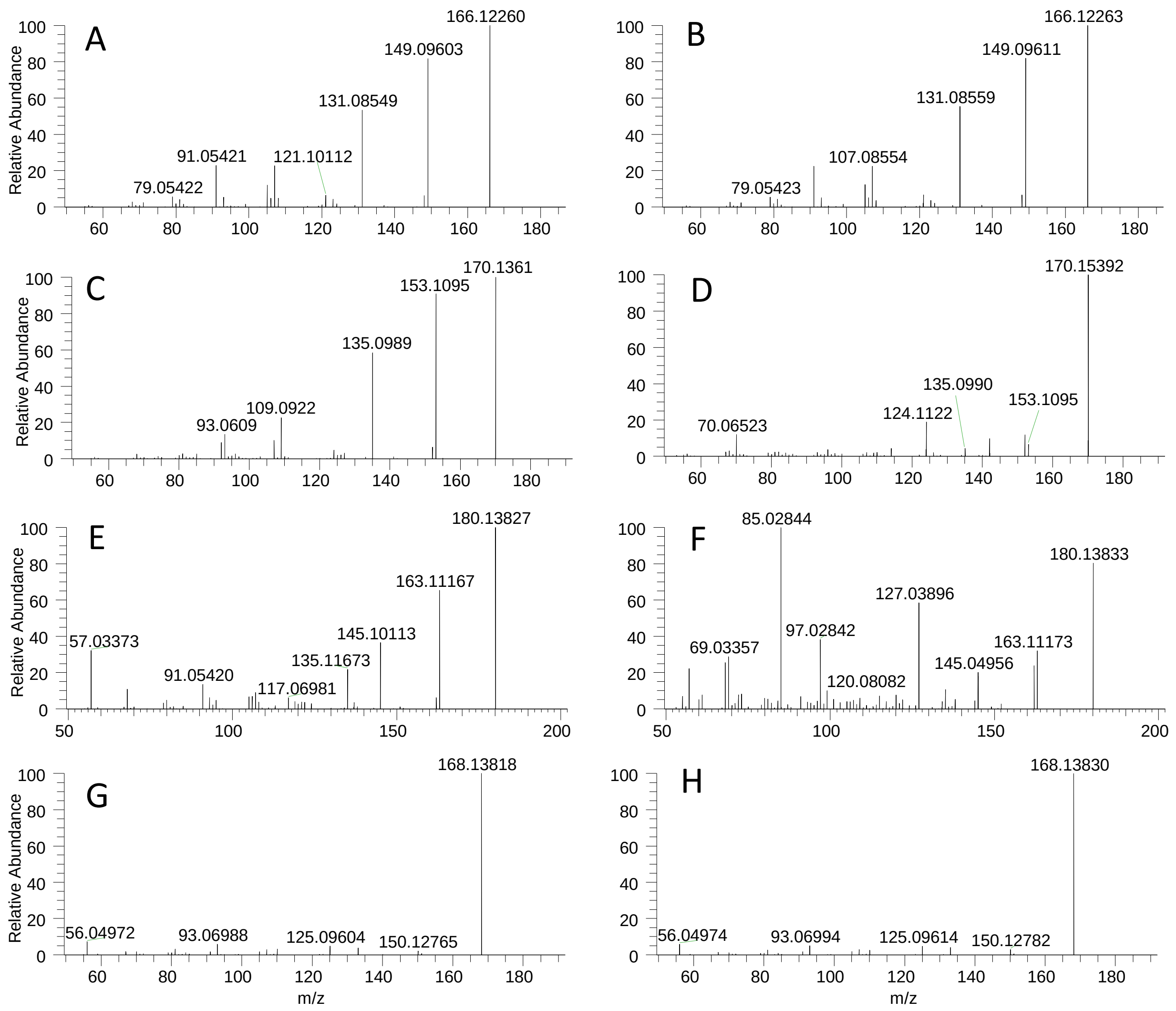

Figure S4: DART-HRMS/MS spectra collected at CE = 15 V of ATX (A, B), $^{13}C_4$-ATX (C,D) hATX (E,F) and H_2-ATX (F,G) detected in a mixed standard (A, C, E, G) or naturally occurring a benthic cyanobacterial mat field sample (B, F, H). Pane D shows detection of $^{13}C_4$-ATX spiked at 60 ng/mL into a cyanobacterial mat lysate sample.

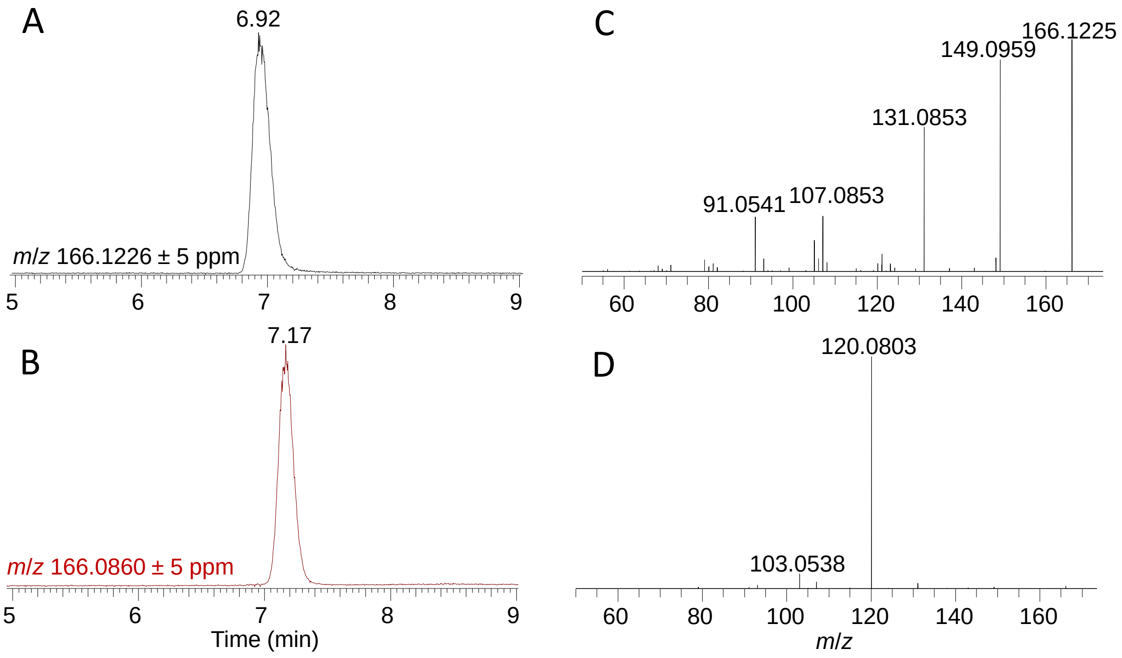

Figure S5: LC-HRMS/MS analysis of a cyanobacterial mat field sample from the Wolastoq showing extracted ion chromatograms (A, B) and product ion spectra (C, D) of ATX (A, C) and phenylalanine (B, D).

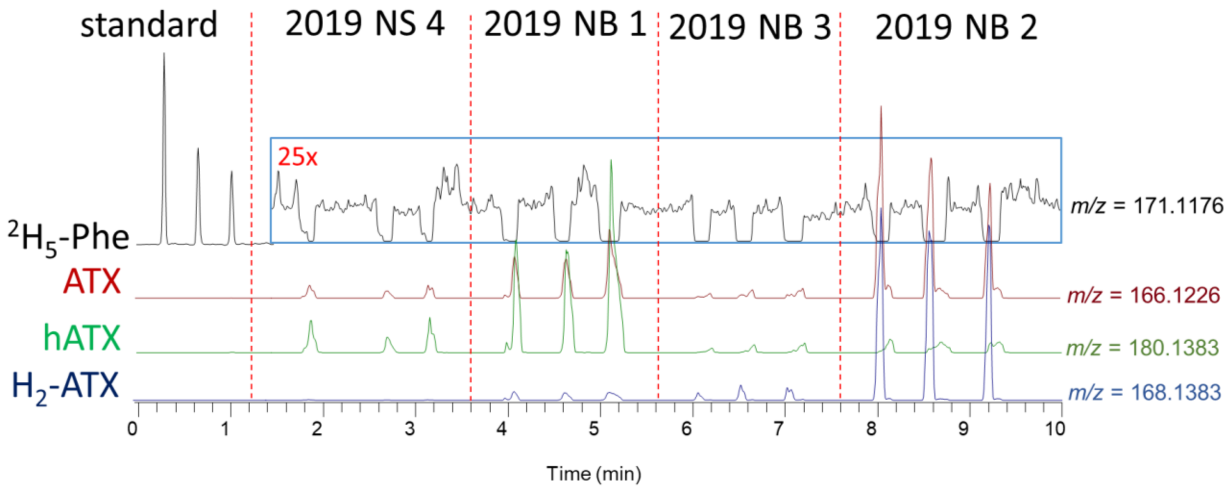

Figure S6: Extracted ion chronograms (± 5 ppm of the *m/z* annotations) of triplicate DART-HRMS analysis of a 100 ng/mL $^2H_5$-Phe standard and cyanobacterial mat field sample extracts spiked with 100 ng/mL $^2H_5$-Phe.

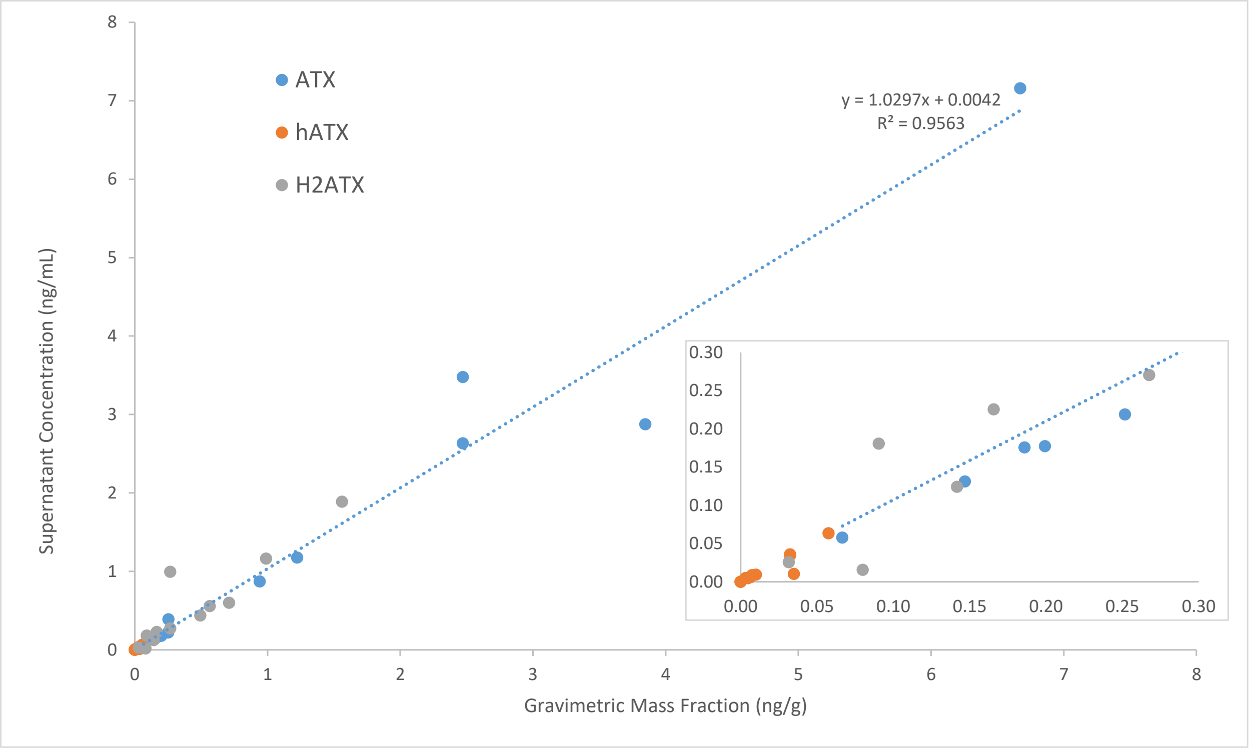

Figure S7: Equivalence of anatoxins measured in 15 benthic cyanobacterial mat samples analyzed in ng/mL and ng/g by LC-HRMS.
